# Supplementary material for: MetaRibo-Seq measures translation in microbiomes
Source: Nat Commun. 2020 Jun 29;11:3268. doi: 10.1038/s41467-020-17081-z (PMC7324362; doi:10.1038/s41467-020-17081-z)
Supplement: Supplementary file 10 — Supplementary Data 7 [file 41467_2020_17081_MOESM10_ESM.zip › File2/Confidence_High_Taxonomy/393205_out.krona.html]

Javascript must be enabled to view this page.

members
magnitude
magnitudeUnassigned
count
unassigned
taxon
rank

393205\_out

3

superkingdom
2
3

1239
3
phylum


SRS104912\_contig\_number\_13958SRS144362\_contig\_number\_36106
1263000
2
species

1
186801
class

order
186802
1


SRS140492\_contig\_number\_25036
species
1
1898207
